# Dietary Leucine Supplementation Improves the Mucin Production in the Jejunal Mucosa of the Weaned Pigs Challenged by Porcine Rotavirus

**DOI:** 10.1371/journal.pone.0137380

**Published:** 2015-09-03

**Authors:** Xiangbing Mao, Minghui Liu, Jun Tang, Hao Chen, Daiwen Chen, Bing Yu, Jun He, Jie Yu, Ping Zheng

**Affiliations:** 1 Animal Nutrition Institute, Sichuan Agricultural University, Ya’an, Sichuan, People’s Republic of China; 2 Key Laboratory of Animal Disease-Resistance Nutrition, Ministry of Education, China, Ya’an, Sichuan, People’s Republic of China; Indian Institute of Science, INDIA

## Abstract

The present study was mainly conducted to determine whether dietary leucine supplementation could attenuate the decrease of the mucin production in the jejunal mucosa of weaned pigs infected by porcine rotavirus (PRV). A total of 24 crossbred barrows weaned at 21 d of age were assigned randomly to 1 of 2 diets supplemented with 1.00% L-leucine or 0.68% L-alanine (isonitrogenous control) for 17 d. On day 11, all pigs were orally infused PRV or the sterile essential medium. During the first 10 d of trial, dietary leucine supplementation could improve the feed efficiency (*P* = 0.09). The ADG and feed efficiency were impaired by PRV infusion (*P*<0.05). PRV infusion also increased mean cumulative score of diarrhea, serum rotavirus antibody concentration and crypt depth of the jejunal mucosa (*P*<0.05), and decreased villus height: crypt depth (*P* = 0.07), goblet cell numbers (*P*<0.05), mucin 1 and 2 concentrations (*P*<0.05) and phosphorylated mTOR level (*P*<0.05) of the jejunal mucosa in weaned pigs. Dietary leucine supplementation could attenuate the effects of PRV infusion on feed efficiency (*P* = 0.09) and mean cumulative score of diarrhea (*P* = 0.09), and improve the effects of PRV infusion on villus height: crypt depth (*P* = 0.06), goblet cell numbers (*P*<0.05), mucin 1 (*P* = 0.08) and 2 (*P* = 0.07) concentrations and phosphorylated mTOR level (*P* = 0.08) of the jejunal mucosa in weaned pigs. These results suggest that dietary 1% leucine supplementation alleviated the decrease of mucin production and goblet cell numbers in the jejunal mucosa of weaned pigs challenged by PRV possibly via activation of the mTOR signaling.

## Introduction

The mucosal barrier of intestine is the first defense line against the luminal hostile environment [1]. The maintenance of intestinal mucosa function mainly depends on the mucosal barrier of gastrointestinal tract that consists of non-specific barrier mechanisms, specific immunological responses and intestinal microecology [2–5]. The mucin-type glycoproteins or mucins that are mainly synthesized and secreted by the goblet cells in the intestinal mucosa are the important component of non-specific barrier mechanisms [6].

Rotavirus is a kind of double-stranded RNA icosahedral RNA virus [7]. It is a major pathogen inducing severe gastroenteritis and diarrhea in children and the other young animals [7, 8]. This could be due to rotavirus damaging the mucosal barrier of the proximal small intestine, including the decrease of the mucin production and the goblet cell number [9–12].

Mucins, a kind of glycoproteins with molecular weights ranging from 0.5 to 20 MDa, consist of the oligosaccharide chains and the protein core [13, 14]. The protein core of small-intestinal mucins in pigs contains a lot of threonine that represents 28–35% of the total amino acid residues [15]. Recent studies have shown that dietary leucine supplementation may decrease serum threonine concentration of pigs [16], and do not affect the total tract apparent threonine digestibility in pigs [17]. Additionally, as a functional amino acid, leucine may regulate protein metabolism in intestines of pigs through the mTOR signaling pathway [16, 18, 19], and stimulate the expression of some specific proteins in tissues and cells [20–23]. Leucine treatment could also inhibit the autophagy via activating the mTOR signaling pathway, which affects the cellular survival and function [24–25]. Thus, it is possible that dietary leucine supplementation stimulated the mucin synthesis in the intestinal mucosa of pigs, and attenuated the effects of rotavirus infection on the mucin production and the goblet cell number of intestinal mucosa. However, these have not been determined.

Therefore, the present study was conducted to test the hypothesis that dietary leucine supplementation could attenuate the decrease of the mucin production in the jejunal mucosa of the weaned pigs infected by porcine rotavirus.

## Materials and Methods

### Animals and diets

The animal protocol for this study was approved by the Animal Care and Use Committee of Sichuan Agricultural University. A total of 24 crossbred (Duroc × Large White × Landrace) barrows, which were weaned at 21 d of age, were housed individually in the metabolism cage (1.5 m × 0.7 m × 1.0 m). The lighting of room was natural, and temperature was maintained at 25–28°C. The pigs were provided with the diets 4 times daily at 0800, 1200, 1600 and 2000 h, and had free access to drinking water. At 0800 h of d 1, 11 and 18, the body weight and feed intake of all pigs were measured, which were used to calculate average daily weight gain (ADG), average daily feed intake (ADFI) and feed conversion. During the experiment, the health of all pigs was monitored every day, and there were not any unexpected deaths.

Two isonitrogenous and isoenergetic diets were formulated to approximately meet National Research Council-recommended nutrient requirements for pigs weighing 5–10 kg (NRC 2012) [26], except for leucine (Table 1). The two diets were supplemented with 1.00% (w/w) L-leucine (Evonik Degussa) or 0.68% (w/w) L-alanine (isonitrogenous control).

**Table 1:** The composition and nutrient content of experimental diets.^#^

	Control	Leu
Ingredients composition, g/kg		
Corn	275.2	275.2
Extruded corn	275.2	275.2
Fish meal	50.0	50.0
Whey powder	30.0	30.0
Soybean meal	130.0	130.0
Soy protein concertrate	45.0	45.0
Extruded full-fat Soybean	120.0	120.0
Corn starch	23.2	20.0
L-Alanine (98.5%)	6.8	0.0
L-Leucine (98.5%)	0.0	10.0
D-Glucose	20.0	20.0
L-Lysine·HCL (78%)	1.9	1.9
L-Threonine (98.5%)	0.5	0.5
DL-Methionine (99%)	1.5	1.5
Choline chloride	1.5	1.5
Sodium Chloride	3.0	3.0
Calcium carbonate	5.4	5.4
Dicalcium phosphate	7.5	7.5
Vitamin premix^1^	0.3	0.3
Mineral premix^2^	3.0	3.0
Nutrient composition, g/kg		
Digestible energy^3^, MJ/kg	14.32	14.32
Crude protein^4^	208.4	207.7
Total lysine^4^	15.8	15.6
Total methionine and cystine^3^	7.4	7.4
Total tryptophan^3^	2.1	2.1
Total threonine^4^	9.0	8.8
Total leucine^4^	17.8	26.2
Calcium^3^	8.1	8.1
Phosphorus available^3^	4.3	4.3

^#^ Control, L-alanine-supplemented (isonitrogenous control) diet; Leu, L-leucine-supplemented diet.

^1^ Provided the following per kg of diet: Vitamin A, 9000 IU; Vitamin D_3_, 3000 IU; Vitamin E, 20 IU; Vitamin K_3_, 3.0 mg; Vitamin B_1_, 1.5 mg; Vitamin B_2_, 4.0 mg; Vitamin B_6_, 3.0 mg; Vitamin B_12_, 0.2 mg; Nicotonic, 30 mg; Pantothenic, 15 mg; Folic acid, 0.75 mg; Biotin, 0.1 mg.

^2^ Provided the following per kg of diet: Fe (as FeSO_4_·7H_2_O), 100 mg; Cu (as CuSO_4_·5H_2_O), 6 mg; Zn (as ZnSO_4_·7H_2_O), 100 mg; Mn (MnSO_4_·H_2_O), 4 mg; Se (as Na_2_SeO_3_·5H_2_O), 0.3 mg; I (as KI), 0.14 mg.

^3^ Calculated nutrient levels.

^4^ Measured nutrient levels.

### Experimental design and sample collection

After 3 d of acclimatization, based on the initial body weights and origin of litters, twenty four piglets were weighed (7.41 ± 0.77) and assigned randomly to 1 of 2 diets supplemented with 1.00% (w/w) L-leucine or 0.68% (w/w) L-alanine (n = 12) for 17 d. On day 11, following orally infused with 5 mL of the sterile 100 mM sodium bicarbonate solution in all piglets, half of the piglets on each diet were orally infused with 4 mL (10^6^ Tissue culture infective dose 50 (TCID_50_)/mL) of procine rotavirus (PRV) dissolved in the essential medium, while the other half were orally infused with the same amount of the sterile essential medium. After PRV infusion, the diarrhea of all pigs was observed. Fecal consistency was scored as follows: 0, normal; 1, pasty; 2, semiliquid; and 3, liquid. The mean cumulative score of diarrhea was calculated as [(∑ fecal scores for 1 week PRV infusion)/n] [27].

On d 18, following weighing, all piglets received their normal diets. At 1.5 h after feeding, the blood samples of 20 mL were obtained by the jugular vein, and centrifuged at 3500 × g for 10 min. The serum was stored at -80°C until analysis. Following the blood sampling, the piglets were killed by an intracardial injection of Na pentobarbital (50 mg/kg body weight) and jugular exsanguinations. Then, the small intestine was removed, and the jejunum (proximal half of the small intestine) was quickly isolated and flushed with ice-cold saline. The segment of the jejunum was collected and fixed in 10% neutral-buffered formalin for the analysis of histomorphology and goblet cells. The tissue of jejunal mucosa was collected through scraping the intestinal wall with a glass microscope slide, frozen in liquid nitrogen, and stored at -80°C until ELISA and Western Blot analysis.

### Porcine rotavirus preparation and virus titre determination

PRV used in this study was a tissue culture-adapted Ohio State University (OSU) strain (ATCC #VR-893). The virus was propagated in the IPEC-J2 cell line, a generous gift from Professor Per Torp Sangild (Copenhagen University, Danmark), as described previously [28]. Briefly, after the pre-activation with 5 μg/mL trypsin (type IX, Sigma) for 30 min at 37°C, the PRV was used to inoculate the IPEC-J2 cell. Following 1 h of incubation at 37°C, the inoculation were removed. IPEC-J2 cells were washed three times with sterile PBS, then incubated at 37°C in Dulbecco’s modified Eagle’s medium/Ham’s F-12 medium (DMEM/F12 medium). When the extensive cytopathic effect (cell destruction) was observed with microscope, the cultures were frozen and thawed three times, and centrifuged at 3000 × g for 10 min. The supernatants containing the PRV were stored at -80°C.

The virus titre (TCID_50_ value) was determined as described previously [29]. Briefly, IPEC-J2 cells were grown to 80–90% confluence in ninety-six-well plates, and then infected with 50 μL aliquots of 1:10 serial dilutions (in DMEM/F12 medium) of PRV samples (8 wells/dilution). Following the incubation for 4 d at 37°C in 5% CO_2_, the cytopathic effect was visualized by staining the remaining viable cells with crystal violet. The virus titres were calculated with the Speaman method (Speaman 1908) and expressed as log_10_ (TCID_50_) [30].

### Chemical analyses

The crude protein of diets was determined according to the Association of Official Analytical Chemists (AOAC 1995) method (AOAC Method 988.05) [31]. The concentrations of amino acids in diets were determined following hydrolyzing in 6 N HCl at 110°C for 24 h (AOAC method 994.12) [31]. Amino-acid analyses were performed by using an L-8800 Amino Acid Analyzer (Hitachi, Tokyo, Japan). The concentrations of plasma free amino acids were also determined using an L-8800 Amino Acid Analyzer (Hitachi, Tokyo, Japan), as previously described by Mao et al. (2014) [32]. Serum urea was measured by an assay kit from Nanjing Jiancheng Biochemistry (Nanjing, China) according to the manufacturer’s instructions.

### Analysis of the rotavirus antibody concentration of the serum and the mucin1 and 2 concentrations of the jejunum

Serum rotavirus antibody concentration was measured by using the commercially available pig enzyme-linked immunosorbent assay (ELISA) kit from TSZ ELISA (Framingham, MA) according to the manufacturer’s instructions. The mucin 1 and 2 in the jejunal mucosa were determined using the commercially available pig mucin 1 and 2 ELISA kits from CUSABIO Biotech Co. Ltd. (China) according to the manufacturer’s instructions. The serum rotavirus antibody and the mucin 1 and 2 in the jejunal mucosa were quantified by using a BioTek Synergy HT microplate reader (BioTek Instruments, Winooski, VT), and absorbance was measured at 450 nm.

### Analysis of the histomorphology and goblet cells in the jejunum

The histomorphology and the count of goblet cells in the jejunum was determined as described previously [33, 34]. Briefly, following the fixing, the segment of the jejunum was embedded in paraffin. Consecutive sections (5 μm) were stained with hematoxylin-eosin and Periodic Acid Schiff- Alcian Blue (PAS-AB) for histomorphological examination and counting goblet cells, respectively. The villus height and crypt depth of the jejunal mucosa was measured at 40 × magnification with an Olympus CK 40 microscope (Olympus Optical Company). The number of positively stained goblet cells was counted within 10 randomly selected villi using Image-Pro Plus software, version 6.0 (Media Cybernetics).

### Western Blot analysis

The antibody against phosphorylated mTOR (Ser^2448^) was purchased from Cell Signaling (Davers, MA). The antibody against β-actin was purchased from Santa Cruz Biotechnology Inc. (Santa Cruz, CA). Protein levels for the phosphorylated mTOR and β-actin in the jejunal mucosa were determined by Western Blot analysis as described previously [22, 23].

### Statistical analysis

All data were expressed as mean ± standard error. Data for the growth performance of pigs during the first 10 d of trial were analyzed using the unpaired *t* test. The other data were analyzed as a 2 × 2 factorial with the general linear model procedures of the SAS (Version 8.1; SAS Institute, Gary, NC). The factors of models included the main effects of leucine treatment (supplemented or unsupplemented with leucine in the diet) and PRV challenge (PRV or sterile essential medium) as well as their interaction. All analyses were performed using SAS (Version 8.1; SAS Institute, Gary, NC). *P*<0.05 was considered to indicate statistical significance, and *P*<0.10 was considered to indicate statistical tendency.

## Results

### The effect of dietary leucine supplementation and/or PRV infusion on the growth performance and diarrhea in the weaned pigs

During the first 10 d of trial, supplementing 1.0% leucine in the diet could improve the feed efficiency (*P* = 0.09), and had no significant effect on the ADG and ADFI of the weaned pigs (Table 2). After PRV infusion, dietary leucine supplementation also improved the feed efficiency (*P* = 0.05), and decreased the mean cumulative score of diarrhea (*P*<0.05) in the weaned pigs (Table 2). However, PRV infusion impaired the ADG and feed efficiency (*P*<0.05), and increased the mean cumulative score of diarrhea (*P*<0.05) in the weaned pigs (Table 2). In addition, dietary leucine supplementation attenuated the effect of PRV infusion on the feed efficiency (*P* = 0.09) and mean cumulative score of diarrhea (*P* = 0.09) in the weaned pigs (Table 2).

**Table 2:** The effect of dietary leucine supplementation and/or PRV infusion on the growth performance and diarrhea in the weaned piglets.^#^

	- PRV		+ PRV		*P*		
	Control	Leu	Control	Leu	Leu	PRV	Leu × PRV
1–10 d							
ADG (g)	165 ± 12	177 ± 9			0.35		
ADFI (g)	315 ± 20	293 ± 12			0.44		
Feed efficiency (g feed/g gain)	1.97 ± 0.13	1.68 ± 0.09			0.09		
11–17 d							
ADG (g)	348 ± 23^a^	344 ± 16^a^	240 ± 31^b^	301 ± 21^ab^	0.24	< 0.05	0.19
ADFI (g)	480 ± 25	464 ± 25	435 ± 30	447 ± 13	0.94	0.22	0.55
Feed efficiency (g feed/g gain)	1.39 ± 0.03^b^	1.35 ± 0.03^b^	1.87 ± 0.16^a^	1.50 ± 0.09^b^	0.05	< 0.05	0.09
The mean cumulative score of diarrhea	4.75 ± 0.75^b^	3.50 ± 0.87^b^	8.50 ± 0.65^a^	5.00 ± 0.41^b^	< 0.05	< 0.05	0.09

^#^ - PRV, infusing the essential medium; + PRV, infusing the procine rotavirus; Control, L-alanine-supplemented (isonitrogenous control) diet; Leu, L-leucine-supplemented diet; ADG, average daily weight gain; ADFI, average daily feed intake.

^a, b^ Mean values within a row with unlike superscript letters were significantly different (*P*<0.05).

### The effect of dietary leucine supplementation and/or PRV infusion on the serum concentrations of free amino acids and urea in the weaned pigs

Dietary 1% leucine supplementation could increase the serum leucine concentration (*P*<0.05), and decrease the serum concentrations of threonine, valine, isoleucine, methionine, phenylalanine, lysine, histidine and urea (*P*<0.05) of the piglets (Table 3). However, PRV infusion increased the serum concentrations of threonine (*P*<0.05), valine (*P*<0.05), isoleucine (*P*<0.05), phenylalanine (*P*<0.05), lysine (*P*<0.05), urea (*P*<0.05) and histidine (*P* = 0.06) of the piglets (Table 3). In addition, dietary leucine supplementation might attenuate the effect of PRV infusion on the serum concentrations of threonine (*P* = 0.09), phenylalaine (*P* = 0.07), lysine (*P* = 0.08), histidine (*P* = 0.09) and urea (*P* = 0.08) in the weaned pigs (Table 3).

**Table 3:** The effect of dietary leucine supplementation and/or PRV infusion on the serum concentrations of free amino acids and urea in the weaned piglets.^#^

	- PRV		+ PRV		*P*		
	Control	Leu	Control	Leu	Leu	PRV	Leu × PRV
Serum free amio acids (μmol/L)							
Leucine	130 ± 7^c^	189 ± 10^a^	150 ± 3^bc^	175 ± 13^ab^	< 0.05	0.73	0.08
Alanine	855 ± 43^a^	561 ± 27^b^	860 ± 61^a^	613 ± 42^b^	< 0.05	0.53	0.61
Threonine	195 ± 19^b^	136 ± 5^c^	266 ± 30^a^	167 ± 26^bc^	< 0.05	< 0.05	0.09
Valine	197 ± 12^b^	134 ± 8^c^	238 ± 14^a^	154 ± 9^c^	< 0.05	< 0.05	0.36
Isoleucine	139 ± 12^a^	84 ± 6^b^	158 ± 6^a^	98 ± 6^b^	< 0.05	< 0.05	0.75
Methionine	64 ± 4^a^	39 ± 4^b^	74 ± 4^a^	42 ± 4^b^	< 0.05	0.13	0.46
Phenylalanine	78 ± 6^bc^	65 ± 4^c^	99 ± 3^a^	82 ± 5^b^	< 0.05	< 0.05	0.07
Lysine	102 ± 8^b^	78 ± 3^b^	151 ± 18^a^	90 ± 4^b^	< 0.05	< 0.05	0.08
Histidine	57 ± 7^b^	16 ± 3^c^	81 ± 12^a^	21 ± 3^c^	< 0.05	0.06	0.09
Serum urea (mmol/L)	2.98 ±0.11^b^	2.22 ± 0.15^c^	3.81 ± 0.13^a^	2.89 ± 0.07^c^	< 0.05	< 0.05	0.08

^#^ - PRV, infusing the essential medium; + PRV, infusing the procine rotavirus; Control, L-alanine-supplemented (isonitrogenous control) diet; Leu, L-leucine-supplemented diet.

^a, b, c^ Mean values within a row with unlike superscript letters were significantly different (*P*<0.05).

### The effect of dietary leucine supplementation and/or PRV infusion on the serum rotavirus antibody in the weaned pigs

PRV infusion increased the serum rotavirus antibody (*P*<0.05), but dietary leucine supplementation had no significant effect on the serum rotavirus antibody in the weaned pigs (Table 4). Furthermore, there was no interaction between dietary leucine supplementation and PRV infusion with regard to the serum rotavirus antibody in the weaned pigs (Table 4).

**Table 4:** The effect of dietary leucine supplementation and/or PRV infusion on the serum rotavirus antibody in the weaned piglets.^#^

	- PRV		+ PRV		*P*		
	Control	Leu	Control	Leu	Leu	PRV	Leu × PRV
Serum rotavirus antibody (ng/L)	3.27 ± 0.73^b^	3.12 ± 0.12^b^	6.61 ± 0.51^a^	6.97 ± 1.02^a^	0.88	< 0.05	0.71

^#^ - PRV, infusing the essential medium; + PRV, infusing the procine rotavirus; Control, L-alanine-supplemented (isonitrogenous control) diet; Leu, L-leucine-supplemented diet.

^a, b^ Mean values within a row with unlike superscript letters were significantly different (*P*<0.05).

### The effect of dietary leucine supplementation and/or PRV infusion on the morphology of the jejunal mucosa in the weaned pigs

Supplementing 1% leucine in the diet reduced the crypt depth (*P*<0.05), and enhanced the villus height (*P*<0.05) and villus height: crypt depth (*P* = 0.09) of the jejunal mucosa in the weaned pigs (Table 5). However, PRV infusion increased the crypt depth (*P*<0.05), and decreased the villus height: crypt depth (*P* = 0.07) of the jejunal mucosa in the weaned pigs (Table 5). In addition, dietary leucine supplementation might attenuate the effect of PRV infusion on the villus height (*P* = 0.07) and villus height: crypt depth (*P* = 0.06) of the jejunal mucosa in the weaned pigs (Table 5).

**Table 5:** The effect of dietary leucine supplementation and/or PRV infusion on the morphology and number of goblet cells in the jejunum of the weaned piglets.^#^

	- PRV		+ PRV		*P*		
	Control	Leu	Control	Leu	Leu	PRV	Leu × PRV
Villus height (μm)	298.84 ± 13.03^ab^	295.31 ± 27.71^ab^	280.78 ± 19.90^b^	357.60 ± 20.48^a^	0.09	0.30	0.07
Crypt depth (μm)	254.52 ± 10.69^b^	213.95 ± 18.94^b^	325.16 ± 20.95^a^	258.98 ± 17.46^b^	< 0.05	< 0.05	0.47
Villus height: crypt depth	1.17 ± 0.01^a^	1.39 ± 0.09^a^	0.88 ± 0.10^b^	1.39 ± 0.06^a^	< 0.05	0.07	0.06
Goblet cells (n/villus)	15 ± 1^a^	18 ± 1^a^	9 ± 2^b^	17 ± 1^a^	< 0.05	< 0.05	< 0.05

^#^ - PRV, infusing the essential medium; + PRV, infusing the procine rotavirus; Control, L-alanine-supplemented (isonitrogenous control) diet; Leu, L-leucine-supplemented diet.

^a, b^ Mean values within a row with unlike superscript letters were significantly different (*P*<0.05).

### The effect of dietary leucine supplementation and/or PRV infusion on the number of goblet cells and the concentrations of mucin 1 and 2 in the jejunal mucosa of the weaned pigs

Dietary 1% leucine supplementation could increase the number of goblet cells (*P*<0.05, Table 5) and the mucin 1 and 2 concentrations (*P*<0.05, Table 6) in the jejunal mucosa of the weaned pigs. However, PRV infusion decreased the number of goblet cells (*P*<0.05, Table 5) and the mucin 1 and 2 concentrations (*P*<0.05, Table 6) in the jejunal mucosa of the weaned pigs. In addition, dietary leucine supplementation attenuated the effect of PRV infusion on the number of goblet cells (*P* < 0.05, Table 5) and the mucin 1 (*P* = 0.08, Table 6) and 2 (*P* = 0.07, Table 6) concentrations in the jejunal mucosa of the weaned pigs.

**Table 6:** The effect of dietary leucine supplementation and/or PRV infusion on the level of mucin 1 and 2 in the jejunal mucosa of the weaned piglets.^#^

	- PRV		+ PRV		*P*		
	Control	Leu	Control	Leu	Leu	PRV	Leu × PRV
Mucin 1 (U/mg protein)	81.81 ± 3.05^b^	110.26 ± 7.83^a^	52.40 ± 2.51^c^	78.96 ± 9.95^b^	< 0.05	< 0.05	0.08
Mucin 2 (ng/mg protein)	10.12 ± 1.48^ab^	12.24 ± 0.81^a^	6.92 ± 0.58^b^	9.85 ± 1.13^ab^	< 0.05	< 0.05	0.07

^#^ - PRV, infusing the essential medium; + PRV, infusing the procine rotavirus; Control, L-alanine-supplemented (isonitrogenous control) diet; Leu, L-leucine-supplemented diet.

^a, b, c^ Mean values within a row with unlike superscript letters were significantly different (*P*<0.05).

### The effect of dietary leucine supplementation and/or PRV infusion on the phosphorylated mTOR level of the jejunal mucosa in the weaned pigs

**Fig 1:**
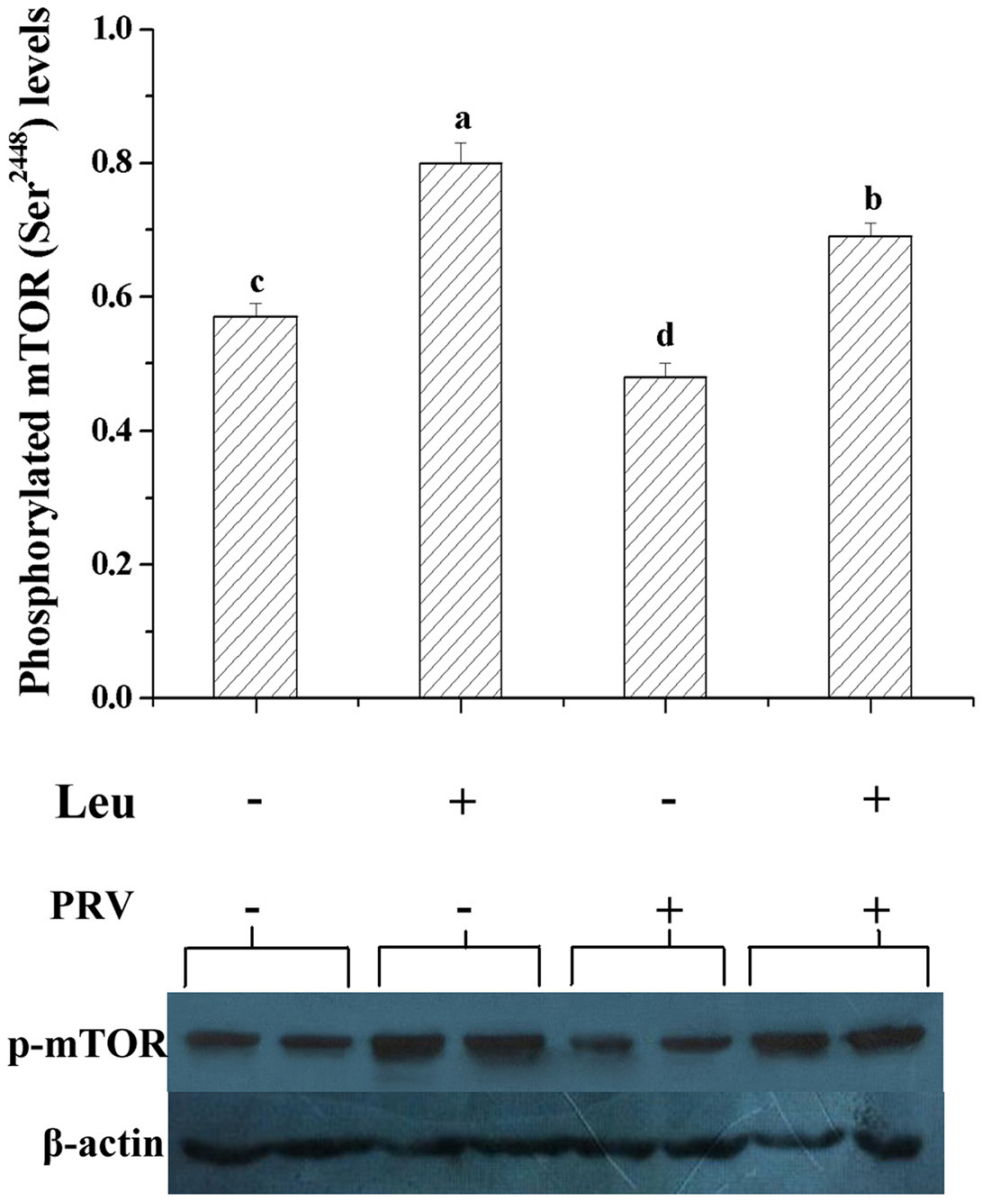
The effect of dietary leucine supplementation and/or PRV infusion on the protein level of the phosphorylated mTOR (p-mTOR) in the jejunal mucosa of the weaned pigs. Representative Western Blots are shown, and results are expressed as the amount of the phosphorylated mTOR relative to β-actin in each group. Values are means (n = 6), with their standard errors represented by vertical bars. ^a, b, c, d^ Mean values with unlike letters were significantly different (*P*<0.05).

Supplementing 1% leucine in the diet could increase the phosphorylated mTOR level of the jejunal mucosa in the weaned pigs (*P*<0.05, Fig 1). However, the phosphorylated mTOR level of the jejunal mucosa was decreased by PRV infusion in the weaned pigs (*P*<0.05, Fig 1). In addition, dietary leucine supplementation attenuated the effect of PRV infusion on the phosphorylated mTOR level of the jejunal mucosa in the weaned pigs (*P* = 0.08, Fig 1).

## Discussion

Rotavirus is one of the main pathogens that lead to the symptomatic gastroenteritis in the young animals, as well as in the young children [7, 8]. Recent study of our lab has shown that PRV challenge decreased the growth performance, impaired the intestinal morphology, and increased the diarrhea in the pigs [35], as well rotavirus could decrease the mucin levels and goblet cell numbers in the intestinal mucosa of the mice [9], which was consistent with the present study (Tables 2, 5 and 6). In addition, this study showed that PRV challenge increased the serum rotavirus antibody in the weaned pigs (Table 4). These results indicated that PRV challenge model was successful.

It has previously been shown that moderate levels of leucine in diets can improve the growth performance of the weaned pigs, but the excess of dietary leucine depress the feed intake and limit growth [16, 18, 36, 37]. In this study, dietary 1% leucine supplementation could improve the feed efficiency of weaned pigs under the normal and PRV challenge conditions (Table 2). Thus, it is clear that the effect of the similar leucine supplementing level on the growth performance of weaned pigs is different among these studies, which could be derived from the differences in the content of leucine and/or protein of diets.

The morphology is important to maintain the normal intestinal function, especially digestive and absorptive function [38]. This study showed that dietary leucine supplementation could improve the morphology, and attenuate the effect of PRV challenge on the morphology in the jejunum (Table 5). These determined that, under normal and PRV challenge conditions, leucine treatment increased the digestion and absorption of nutrients in the intestine of pigs, which could be an important reason that supplementing 1% leucine in diets improved the growth performance and decreased the diarrhea.

The blood concentrations of free amino acids and urea could indirectly indicate the amount of amino acids that are available for tissue growth [39]. This study also showed that PRV challenge increased the serum concentrations of free amino acids and urea in the weaned pigs (Table 3), which determined that PRV challenge could impair the protein metabolism of whole body. It is possible that PRV inducing the decrease of growth performance partially derived from the protein metabolic disturbance. However, recent studies have shown that oral leucine administration may significantly affect the serum or plasma levels of the free amino acids in rats, mice and pigs [16, 18, 23, 40–43], which is similar with the present study (Table 3). In this study, dietary leucine supplementation also attenuated, to some extent, the effect of PRV challenge on the blood concentrations of free amino acids and urea in the weaned pigs (Table 3), which could determine that supplementing leucine in diets attenuated the protein metabolic disturbance induced by PRV challenge, and then possibly improved the growth performance.

Some studies with humans and pigs have shown that 20–70% of the first-pass metabolism of essential amino acids in diets is consumed by the portal-drained viscera (PDV), and a large amount of dietary threonine (40–60%) were extracted by the PDV (dominated by the intestinal mucosa) in the first pass metabolism [44, 45]. In addition, the peptide backbone of small-intestinal mucins in pigs contains a lot of threonine that represents 28–35% of the total amino acid residues [46]. The present study also showed that, under normal and PRV challenge conditions, dietary leucine supplementation decreased serum threonine concentration of pigs at 1.5 h after feeding (Table 3), which is consistent with the result of the previous studies [16, 23, 40–43]. However, this study showed that supplementing 1% leucine in diets could increase the mucin 1 and 2 concentrations of mucosa (Table 6).

Mucins mainly synthesized and secreted by the goblet cells are the important component of non-specific barrier mechanisms in the intestinal mucosa, which will protect animals and humans against enteric pathogens [6]. Recent studies have shown that rotavirus damages the mucosal barrier function of the proximal small intestine, including decreasing the mucin levels via destroying the goblet cells, which is one of the reasons that rotavirus leads to the diarrhea [9–12]. Thus, in this study, dietary 1% leucine supplementation attenuate the effect of PRV challenge on the mucin 1 and 2 concentration and the goblet cell number in the jejunal mucosa of weaned pigs (Tables 2, 5 and 6), which could be the reason that leucine treatment decreased the diarrhea induced by the PRV infection.

In the cell or tissue, the mTOR signaling is one of the most important pathways regulating the protein metabolism and the autophagy that may directly affect the cellular function and survival through exerting both beneficial and aggravating effects on the progression of disease [47, 48]. In the present study, PRV challenge decreased the phosphorylated mTOR level in the jejunal mucosa of weaned pigs (Fig 1), which could impair the mucin synthesis and the survival of the goblet cells. Thus, it is possible that rotavirus decreasing the mucin production was relative with inhibition of the mTOR signaling pathway.

Recent studies have shown that dietary leucine supplementation may stimulate the mTOR signaling and the protein synthesis in the intestine of piglets [16, 18], and enhance the production of specific proteins in various tissues and cells through stimulating the mTOR signaling [20–23]. Moreover, oral leucine administration can decrease the protein degradation in human duodenum, and increase the mucosal cell proliferation via activating the PI3K/Akt/mTOR signaling [19]. In this study, dietary 1% leucine supplementation also stimulated the phosphorylated mTOR level in the intestinal mucosa of pigs, and even attenuated, to some extent, the effect of PRV challenge on it (Fig 1). Therefore, these indicated that supplementing 1% leucine in diets efficiently alleviated the effect of PRV challenge on the mucin production and the goblet cell number of the jejunal mucosa possibly via stimulating the phosphorylated mTOR level in the goblet cells.

## Conclusion

Rotavirus infection impaired the growth performance of the weaned pigs via the protein metabolic disturbance of whole body and the diarrhea derived from the jejunal mucosa dysfunction. However, dietary 1% leucine supplementation alleviated the effect of rotavirus infection on growth performance and diarrhea in weaned pigs, which could be due that leucine treatment could improve the protein metabolism, the digestive and absorptive function of intestine, and non-specific barrier mechanism of intestinal mucosa. These findings provide a basis for researching the different functions of leucine in animals and humans.

## References

[1] Blikslager AT, Moeser AJ, Gookin JL, Jones SL, Odle J. Restoration of barrier function in injured intestinal mucosa. Physiol Rev. 2007;87: 545–564. 17429041 10.1152/physrev.00012.2006

[2] Chen H, Wang W, Degroote J, Possemiers S, Chen D, De Smet S, et al Arabinoxylan in wheat is more responsible than cellulose for promoting intestinal barrier function in weaned male piglets. J Nutr. 2015;145: 51–58. 10.3945/jn.114.201772 25378684

[3] Farhadi A, Banan A, Fields J, Keshavarzian A. Intestinal barrier: an interface between health and disease. J Gastroen Hepatol. 2003;18: 479–497. 10.1046/j.1440-1746.2003.03032.x 12702039

[4] Langkamp-Henken B, Glezer JA, Kudsk KA. Immunologic structure and function of the gastrointestinal tract. Nutr Clin Pract. 1992;7: 100–108. 1289681 10.1177/0115426592007003100

[5] Magalhaes JG, Tattoli I, Girardin SE. The intestinal epithelial barrier: how to distinguish between the microbial flora and pathogens. Semin Immunol. 2007;19: 106–115. 17324587 10.1016/j.smim.2006.12.006

[6] Forstner JF, Forstner GG. Gastrointestinal mucus In: Johnson LR, editors. Physiology of the gastrointestinal tract. 3rd ed New York: Raven Press; 1994 pp. 1255–1283.

[7] Donelli G, Superti F. The rotavirus genus. Comp Immunol Microbiol Infect Dis. 1994;17: 305–320. 8001352 10.1016/0147-9571(94)90050-7

[8] Barnett B. Viral gastroenteritis. Med Clin North Am. 1983;67: 1031–1058. 6312212 10.1016/S0025-7125(16)31165-8 PMC7130486

[9] Boshuizen JA, Reimerink JHJ, Korteland-van Male AM, van Ham VJJ, Bouma J, Gerwig GJ, et al Homeostasis and function of goblet cells during rotavirus infection in mice. Virology. 2005;337: 210–221. 15882887 10.1016/j.virol.2005.03.039

[10] Arnold MM, Patton JT. Diversity of interferon antagonist activities mediated by NSP1 proteins of different rotavirus strains. J Virol. 2011;85: 1970–1979. 10.1128/JVI.01801-10 21177809 PMC3067804

[11] Pott J, Mahlakoiv T, Mordstein M, Duerr CU, Michiels T, Stockinger S, et al IFN-λ determines the intestinal epithelial antiviral host defense. Proc Natl Acad Sci U S A. 2011;108: 7944–7949. 10.1073/pnas.1100552108 21518880 PMC3093475

[12] Wu S, Yuan L, Zhang Y, Liu F, Li G, Wen K, et al Probiotic Lactobacillus rhamnosus GG mono-association suppresses human rotavirus-induced autophagy in the gontobiotic piglet intestine. Gut Pathog. 2013;5: 22 10.1186/1757-4749-5-22 23924832 PMC3750464

[13] Bansil R, Turner BS. Mucin structure, aggregation, physiological functions and biomedical applications. Curr Opin Colloid Interf Sci. 2006;11: 164–170.

[14] Sasaki M, Ikeda H, Nakanuma Y. Expression profiles of MUC mucins and trefoil factor family (TFF) peptides in the intrahepatic biliary system: physiological distribution and pathological significance. Prog Histochem Cyto. 2007;42: 61–110. 10.1016/j.proghi.2007.02.001 17616258

[15] Mantle M, Allen A. Isolation and characterization of the native glycoprotein from pig small-intestinal mucus. Biochem J. 1981;195: 267–275. 7306053 10.1042/bj1950267 PMC1162882

[16] Yin Y, Yao K, Liu Z, Gong M, Ruan Z, Deng D, et al Supplementing L-leucine to a low-protein diet increases tissue protein synthesis in weanling pigs. Amino Acids. 2010;39: 1477–1486. 10.1007/s00726-010-0612-5 20473536

[17] Chu L. Study on effects of leucine supplementation in low crude protein diets on protein metabolism of adult rats and finishing pigs. PhD Dissertation, China Agricultural University. 2012.

[18] Torrazza RM, Suryawan A, Gazzaneo MC, Orellana RA, Frank JW, Nguyen HV, et al Leucine supplementation of a low-Protein meal increases skeletal muscle and visceral tissue protein synthesis in neonatal pigs by stimulating mTOR-dependent translation initiation. J Nutr. 2010;140: 2145–2152. 10.3945/jn.110.128421 20962152 PMC2981001

[19] Coëffier M, Claeyssens S, Bensifi M, Lecleire S, Boukhettala N, Maurer B, et al Influence of leucine on protein metabolism, phosphokinase expression, and cell proliferation in human duodenum. Am J Clin Nutr. 2011;93: 1255–1262. 10.3945/ajcn.111.013649 21508089

[20] Roh C, Han J, Tzatsos A, Kandror KV. Nutrient-sensing mTOR-mediated pathway regulates leptin productin in isolated rat adipocytes. Am J Physiol Endocrinol Metab. 2003;284: E322–E330. 12388166 10.1152/ajpendo.00230.2002

[21] Lynch CJ, Gern B, Lloyd C, Hutson SM, Eicher R, Vary TC. Leucine in food mediates some of the postprandial rise in plasma leptin concentrations. Am J Physiol Endocrinol Metab. 2006;291: E621–E630. 16638821 10.1152/ajpendo.00462.2005

[22] Mao X, Zeng X, Wang J, Qiao S. Leucine promotes leptin receptor expression in mouse C2C12 myotubes through the mTOR pathway. Mol Biol Rep. 2011;38: 3201–3206. 10.1007/s11033-010-9992-6 20151325

[23] Mao X, Zeng X, Huang Z, Wang J, Qiao S. Leptin and leucine synergistically regulate protein metabolism in C2C12 myotubes and mouse skeletal muscles. Br J Nutr. 2013;110: 256–264. 10.1017/S0007114512004849 23211060

[24] Mordier S, Deval C, Béchet D, Tassa A, Ferrara M. Leucine limitation induces autophagy and activation of lysosome-dependent proteolysis in C2C12 myotubes through a mammalian target of rapamycin-independent signaling pathway. J Biol Chem. 2000;275: 29900–29906. 10893413 10.1074/jbc.M003633200

[25] Dodd KM, Tee AR. Leucine and mTORC1: a complex relationship. Am J Physiol Endocrinol Metab. 2012;302: E1329–E1342. 10.1152/ajpendo.00525.2011 22354780

[26] National Research Council. Nutrient Requirements of Swine. 10th ed Washington, DC: National Academy Press 1998.

[27] Yuan L, Kang SY, Ward LA, To TL, Saif LJ. Antibody-secreting cell responses and protective immunity assessed in gnotobiotic pigs inoculated orally or intramuscularly with inactivated human rotavirus. J Virol. 1998;72: 330–338. 9420231 10.1128/jvi.72.1.330-338.1998 PMC109380

[28] Liu F, Li G, Wen K, Bui T, Cao D, Zhang Y, et al Porcine small intestinal epithelial cell line (IPEC-J2) of rotavirus infection as a new model for the study of innate immune responses to rotaviruses and probiotics. Viral Immunol. 2010;23: 135–149. 10.1089/vim.2009.0088 20373994 PMC2883522

[29] Botić T, Klingberg TD, Weingartl H, Cencic A. A novel eukaryotic cell culture model to study antiviral activity of potential probiotic bacteria. Int J Food Microbiol. 2007;115: 227–234. 17261339 10.1016/j.ijfoodmicro.2006.10.044

[30] Spearman C. The method of right and wrong cases (constant stimuli) without Gauss’s formulae. Br J Psychol. 1908;2: 227–242.

[31] Association of Official Analytical Chemists. Official Methods of Analysis. 16th ed Arlington, Virginia: Association of Official Analytical Chemists 1995.

[32] Mao X, Lv M, Yu B, He J, Zheng P, Yu J, et al The effect of dietary tryptophan levels on oxidative stress of liver induced by diquat in weaned piglets. J Anim Sci Biotech. 2014;5: 49. 10.1186/2049-1891-5-49 PMC4373006 25810902

[33] Wang W, Zeng X, Mao X, Wu G, Qiao S. Optimal dietary ture ileal digestible threonine for supporting the mucosal barrier in small intestine of weanling pigs. J Nutr. 2010;140: 981–986. 10.3945/jn.109.118497 20335627

[34] Chen H, Mao X, He J, Yu B, Huang Z, Yu J, et al Dietary fibre affects intestinal mucosal barrier function and regulates intestinal bacteria in weaning piglets. Br J Nutr. 2013;110: 1837–1848. 10.1017/S0007114513001293 23656640

[35] Zhao Y, Yu B, Mao X, He J, Huang Z, Zheng P, et al Dietary vitamin D supplementation attenuates immune responses of pigs challenged with rotavirus potentially through the retinoic acid-inducible gene I signalling pathway. Br J Nutr. 2014;112: 381–389. 10.1017/S000711451400097X 24833277

[36] Edmonds MS, Baker DH. Amino acid excesses for young pigs: effects of excess methionine, tryptophan, threonine or leucine. J Anim Sci. 1987;64: 1664–1671. 3110116 10.2527/jas1987.6461664x

[37] Gatnau R, Zimmerman DR, Nissen SL, Wannemuehler M, Ewan RC. Effects of excess dietary leucine and leucine catabolites on growth and immune responses in weanling pigs. J Anim Sci. 1995;73: 159–165. 7601729 10.2527/1995.731159x

[38] Dibner J, Richards J. The digestive system: challenges and opportunities. J Appl Poultry Res. 2004;13: 86–93.

[39] Coma J, Carrion D, Zimmerman DR. Use of plasma urea nitrogen as a rapid response criterion to determine the lysine requirement of pigs. J Anim Sci. 1995;73: 472–481. 7601781 10.2527/1995.732472x

[40] Rieu I, Sornet C, Bayle G, Prugnaud J, Pouyet C, Balage M, et al Leucine-supplemented meal feeding for ten days beneficially affects postprandial muscle protein synthesis in old rats. J Nutr. 2003;133: 1198–1205. 12672943 10.1093/jn/133.4.1198

[41] López N, Sánchez J, Picó C, Palou A, Serra F. Dietary L-leucine supplementation of lactating rats results in a tendency to increase lean/fat ratio associated to lower orexigenic neuropeptide expression in hypothalamus. Peptides. 2010;31: 1361–1367. 10.1016/j.peptides.2010.03.028 20347902

[42] Balage M, Dupont J, Mothe-Satney I, Tesseraud S, Mosoni L, Dardevet D. Leucine supplementation in rats induced a delay in muscle IR/PI3K signaling pathway associated with overall impaired glucose tolerance. J Nutr Biochem. 2011;22: 219–226. 10.1016/j.jnutbio.2010.02.001 20558053

[43] Mao X, Zeng X, Cai C, Ren M, Qiao S. Effect of dietary leucine supplementation on plasma leptin level and protein metabolism of skeletal muscles in rats. Chinese J Anim Sci. 2011;47: 26–30.

[44] Stoll B, Burrin DG, Henry J, Yu H, Jahoor F, Reeds PJ. Substrate oxidation by the portal drained viscera of fed piglets. Am J Physiol Endocrinol Metab. 1999;277: E168–E175. 10.1152/ajpendo.1999.277.1.E168 10409141

[45] Stoll B, Henry J, Reeds PJ, Yu H, Jahoor F, Burrin DG. Catabolism dominates the first-pass intestinal metabolism of dietary essential amino acids in milk protein-fed piglets. J Nutr. 1998;138: 606–614. 10.1093/jn/128.3.606 9482771

[46] Schaart MW, Schierbeek H, Van Der Schoor SR, Stoll B, Burrin DG, Reeds PJ, et al Threonine utilization is high in the intestine of piglets. J Nutr. 2005;135: 765–770. 15795432 10.1093/jn/135.4.765

[47] Sridhar S, Botbol Y, Macian F, Cuervo AM. Autophagy and disease: always two sides to a problem. J Pathol. 2012;226: 255–273. 10.1002/path.3025 21990109 PMC3996449

[48] Goodman CA. The role of mTORC1 in regulating protein synthesis and skeletal muscle mass in response to various mechanical stimuli. Rev Physiol Biochem Pharmacol. 2014;166: 43–95. 10.1007/112_2013_17 24442322

